# Operationalizing the reach, effectiveness, adoption, implementation, maintenance (RE-AIM) framework to evaluate the collective impact of autonomous community programs that promote health and well-being

**DOI:** 10.1186/s12889-019-7131-4

**Published:** 2019-06-24

**Authors:** Robert B. Shaw, Shane N. Sweet, Christopher B. McBride, William K. Adair, Kathleen A. Martin Ginis

**Affiliations:** 1 School of Health & Exercise Sciences, University of British Columbia, 1147 Research Rd, Kelowna, British Columbia V1V 1V7 Canada; 2 International Collaboration on Repair Discoveries (ICORD), University of British Columbia, 818 West 10th Avenue, Vancouver, British Columbia V5Z 1M9 Canada; 3 Department of Kinesiology and Physical Education, McGill University, 475 Pine Ave W, Montreal, Quebec H2W 1S4 Canada; 4 Center for Interdisciplinary Research in Rehabilitation of Greater Montreal, 6363 Chemin Hudson (Pavillon Lindsay) suite 061, Montreal, Quebec H3S 1M9 Canada; 5 Spinal Cord Injury BC, 780 SW Marine Drive, Vancouver, British Columbia V6P 5Y7 Canada; 6 Spinal Cord Injury Canada, 520 Sutherland Drive, Toronto, Ontario M4G 3V9 Canada; 7 Department of Medicine, Division of Physical Medicine & Rehabilitation, University of British Columbia, 2329 West Mall, Vancouver, British Columbia V6T1Z4 Canada

**Keywords:** RE-AIM, Spinal cord injury, Peer mentorship, Peer support, Community

## Abstract

**Background:**

The RE-AIM (Reach, Effectiveness, Adoption, Implementation, and Maintenance) framework is a useful tool for evaluating the impact of programs in community settings. RE-AIM has been applied to evaluate individual programs but seldom used to evaluate the collective impact of community-based, public health programming developed and delivered by multiple autonomous organizations. The purposes of this paper were to (a) demonstrate how RE-AIM can be operationalized and applied to evaluate the collective impact of similar autonomous programs that promote health and well-being and (b) provide preliminary data on the collective impact of Canadian spinal cord injury (SCI) peer mentorship programs on the delivery of peer mentorship services.

**Methods:**

Criteria from all five RE-AIM dimensions were operationalized to evaluate multiple similar community-based programs. For this study, nine provincial organizations that serve people with SCI were recruited from across Canada. Organizations completed a structured self-report questionnaire and participated in a qualitative telephone interview to examine different elements of their peer mentorship program. Data were analyzed using summary statistics.

**Results:**

Having multiple indicators to assess RE-AIM dimensions provided a broad evaluation of the impact of Canadian SCI peer mentorship programs. Peer mentorship programs reached 1.63% of the estimated Canadian SCI population. The majority (67%) of organizations tracked the effectiveness of peer mentorship through testimonials and reports. Setting-level adoption rates were high with 100% of organizations offering peer mentorship in community and hospital settings. On average, organizations allocated 10.4% of their operating budget and 9.8% of their staff to implement peer mentorship and 89% had maintained their programming for over 10 years. Full interpretation of the collective impact of peer mentorship programs was limited as complete data were only collected for 52% of survey questions.

**Conclusions:**

The lack of available organizational data highlights a significant challenge when using RE-AIM to evaluate the collective impact of multiple programs that promote health and well-being. Although researchers are encouraged to use RE-AIM to evaluate the collective impact of programs delivered by different organizations, documenting limitations and providing recommendations should be done to further the understanding of how best to operationalize RE-AIM in these contexts.

**Electronic supplementary material:**

The online version of this article (10.1186/s12889-019-7131-4) contains supplementary material, which is available to authorized users.

## Background

The World Health Organization supports the implementation of community interventions to combat noncommunicable diseases but stresses the importance of monitoring their effectiveness and progress [1]. Monitoring the impact of community-based public health programs, interventions and public services that promote a healthy lifestyle can be a complex task. To fully capture the impact of a program, one needs to evaluate not only the impact on participants, but also the impact on the organization providing a program and the broader community. The RE-AIM planning and evaluation framework is a framework that can be used to comprehensively evaluate both the individual and organizational impact of a program or intervention [2].

Consisting of five evaluation dimensions (Reach, Effectiveness, Adoption, Implementation, Maintenance), RE-AIM has been used effectively across a variety of settings (e.g., community, policy, public health) [3]. The definitions of the five RE-AIM dimensions are intended to be straightforward to enable the framework to be easily understood and applied [4, 5]. Reach represents the absolute number and proportion of individuals who are willing to participate in a given initiative and how representative participants are compared to the target population. Effectiveness describes the impact of an intervention or program on important outcomes, including potential positive and negative effects, quality of life, and economic outcomes. Adoption is the absolute number and proportion of settings and intervention staff who are willing to initiate an intervention or program. Implementation refers to the degree to which the intervention or program staff deliver the initiative as intended as well as the related costs. Implementation also refers to participants’ use of the intervention’s strategies or the program’s services. Lastly, Maintenance refers to the sustained delivery and effectiveness of the initiative.

The RE-AIM framework was originally developed to provide researchers with an evaluation tool that could determine the public health or population-based impact of a program or policy while taking into consideration program indicators relating to both internal and external validity [2, 6, 7]. Over time, the framework has evolved to incorporate several evaluation items for each RE-AIM dimension [3], has been used across diverse content areas [8–10], and has expanded to use as both a planning, and evaluation tool [3]. RE-AIM has been applied to study planning or evaluation in over 450 published studies [11], has been cited in numerous grant proposals [3], and has allowed researchers to evaluate the population health impact of both clinical and community-based interventions.

Typically, RE-AIM has been used by researchers to assess the impact of a single research intervention in achieving behaviour change at the individual level. Within a public health context, the framework has been used to evaluate behaviour change interventions targeting healthy eating [12], physical activity and exercise participation [12, 13], smoking cessation [14], and disease management [15]. Recently, RE-AIM was operationalized successfully to evaluate the impact of a community-based public health initiative delivered in partnership between community organizations and academic researchers [16]. Implementing RE-AIM to evaluate the impact of this partnership not only demonstrated the utility of the framework within a public health context, but also highlighted its value for providing community organizations with information that could directly impact their operations.

Though these examples provide evidence of the utility of the RE-AIM framework to evaluate community-based public and population health interventions, implementing the RE-AIM framework can be challenging. A recent review of 42 National Institutes of Health grant applications determined that only 10% of applications intended to address all five RE-AIM dimensions [17]. Moreover, a recent systematic review found that only 62% of published studies using the RE-AIM framework reported on all five dimensions and not a single study was able to address all 34 criteria [3]. When considered alongside each other, these findings highlight how challenging it can be to perform a complete RE-AIM analysis, even in well-funded research projects, let alone for community-based public health programming delivered by community organizations.

### Applying RE-AIM to evaluate programming developed by community organizations

While the RE-AIM framework has seen an increase in usage in policy, primary care, and public health care settings, its use in community settings has still largely been to evaluate the translation of public health research interventions into real world programming [3]. Seldom has the framework been applied to evaluate real-world community-based public health programming developed and delivered by community organizations. For example, Burke and colleagues [18] applied the framework to evaluate the feasibility of a summer camp that was delivered in part through the Young Men’s Christian Association (YMCA). More recently, Jung, Bourne, and Gainforth [19] applied the framework in its entirety to evaluate a physical activity and healthy eating program delivered across ten different sites by a not-for-profit organization. Although both studies applied RE-AIM in an evaluative capacity, the evaluated programs had first existed as research interventions that were translated into community programming. To our knowledge, Koorts and Gillison’s [20] evaluation of a community-based physical activity program is the only published study to use all five dimensions of the RE-AIM framework to evaluate programming developed and implemented independently by a community organization. Furthermore, we are unaware of any published study that has used the framework to evaluate the collective impact of several autonomous community-based programs (e.g., peer mentorship programs delivered by different spinal cord injury organizations) that have similar health promotion objectives.

The challenges of using the RE-AIM framework in community settings [3, 4, 17, 20] may be exacerbated when using it to evaluate the collective impact of multiple autonomous community-based programs delivered by separate organizations. For example, community organizations, who often encounter funding challenges, may be unable to collect the primary data required to complete a full RE-AIM analysis [4]. This challenge could result in missing or incomplete data across several programs which could limit the analysis to only a few indicators within each RE-AIM dimension. Furthermore, aggregating data to evaluate the collective impact of certain outcomes may not be possible if organizations are collecting slightly different data. Engaging stakeholders from multiple organizations would be necessary to ensure that a) data being collected are similar enough to be aggregated, and b) the measures used in the evaluation are appropriate indicators within the RE-AIM framework [17].

Despite the potential challenges, using the RE-AIM framework to evaluate the collective impact of community-based health promoting programs/initiatives could provide organizations with important information regarding the maintenance and implementation of programming. Although most organizations track basic metrics (e.g., fitness program memberships), greater insight could be gained by evaluating the collective impact of a specific program offered across a variety of organizations (e.g., yoga classes). The framework could also be used to evaluate the collective impact of various health promoting programs for people living with a particular chronic condition.

A specific context where the RE-AIM framework could be beneficial would be the evaluation of peer mentorship programs that promote healthy living for people with spinal cord injury (SCI). Secondary complications associated with SCI contribute largely to the burden placed on the public health system and its services [21]. Long-term health complications, including cardiovascular complications and respiratory problems place strain on hospitals, medical professionals and attendants/care workers. Furthermore, impaired physical functioning following an SCI can complicate obtaining or returning to employment which places additional strain on public/government programs (e.g., disability support programs) [22]. Interventions and public programs that help individuals manage these secondary complications therefore play an important role in reducing the strain on public health care systems.

Community-based peer mentorship programs, offered in the public sector, have potential to reduce the economic impact of SCI. Research has shown that SCI peer mentorship has a positive impact on self-management to prevent secondary conditions [23], can reduce rehospitalization rates [24], and is associated with work/education participation [25]. Across North America, there are several non-profit SCI organizations that offer peer mentorship for community-dwelling individuals living with SCI. Although each organizations’ peer mentorship program is unique, they all share the same mission, to help people with SCI adapt and thrive in the community. Within a SCI context, peer mentorship involves initiating a relationship between a peer mentor (an individual who has successfully faced living with a SCI) with a peer mentee (someone with a SCI who is in need of support). A mentor-mentee relationship can be short-lived or last for years. The mentor, because of their lived experience, is able to provide a wide variety of support types (i.e. emotional, informational, esteem, instrumental) to the mentee. Research examining these programs has focused mainly on the individual level benefits associated with receiving peer mentorship [23, 24, 26]. Interestingly, despite the known individual level benefits of peer mentorship, little is known about the impact of peer mentorship programs at the organizational level. Operationalizing the RE-AIM framework to describe the impact of peer mentorship at the organizational level could provide community organizations with valuable information to inform the creation of new programming and to help maintain existing programs.

The application of the RE-AIM framework to evaluate the collective impact of community-based public health programming addresses the WHO call to monitor the progress of public health interventions [1]. Despite the potential challenges of using the framework in this capacity [4, 15], previous studies have shown its utility as an evaluation tool across various settings and populations [3]. Therefore, given RE-AIM’s utility as an evaluation tool for “real-world” programs, the purposes of this study were to a) operationalize and apply each dimension of the RE-AIM framework to evaluate similar community-based public health programs delivered by multiple, autonomous community organizations and b) present findings regarding the impact of Canadian SCI peer mentorship programs. As the present study is the first to apply the RE-AIM framework in this context, a set of recommendations to inform future research and application of the RE-AIM framework will be provided.

## Methods

### Program selection and recruitment

A purposive sampling method was used to select programs. Recruitment was facilitated by SCI Canada, a not-for-profit organization whose mission is to assist people with SCI and other physical disabilities to achieve independence, self-reliance, and full community participation [27]. SCI Canada’s national network consists of eight autonomous provincial organizations who serve people with SCI as their main clientele. These eight organizations provide their own individualised peer mentorship programs for people with traumatic (e.g., car accidents, falls), non-traumatic (e.g., acquired from disease), and congenital (e.g., spina bifida) SCI in both hospital and community settings. The executive director of SCI Canada contacted the executive directors from each of the eight provincial organizations through email and provided a study information sheet. Additional emails were sent to two provincial disability organizations who do not belong to the SCI Canada national network but who provide similar community-based peer mentorship programming for people with SCI. Organizations that expressed interest were then contacted by the lead researcher who obtained informed consent from the executive director of the organization. Ten organizations, and their executive directors, from ten different provinces provided informed consent and nine completed the study. The one organization that did not complete the study withdrew for unknown reasons.

### Design and operationalization of RE-AIM dimensions

This study used a cross-sectional study design, whereby two surveys were administered to collect data from participating organizations/executive directors. Questions for both surveys were developed by operationalizing indicators within each RE-AIM dimension for SCI peer mentorship. Brief descriptions of how each dimension was operationalized are provided below.

#### Reach

Given that community organizations may aim to reach individuals who may play different roles within a specific program, reach indicators needed to capture all key individuals who play a role in delivering program services or who receive services. Conceptualizing reach indicators for SCI peer mentorship was unique in that individuals who provide (mentor) or receive (mentee) mentorship can be considered “participants”. Therefore, when conceptualizing reach indicators, we included separate questions for both types of participants and collected separate demographic characteristics for each. Further, although the Reach dimension should include data on excluded individuals (e.g., people who contact SCI organizations for peer mentorship but who do not qualify for the service) [3], the stakeholders who co-designed the survey were confident that SCI organizations were not collecting this information due to resource restrictions. Therefore, we did not include this indicator in our survey.

#### Effectiveness

Unlike clinical research interventions, high levels of experimental control and rigour are not practical or feasible for community-based organizations to evaluate their programs (e.g., using pre-post assessments). Rather, community programs may monitor members’ general experiences through testimonials or simplified surveys that do not necessarily include validated measures. The lack of valid and reliable measures would limit a researcher’s ability to accurately report on the effectiveness of a program. In consultation with our stakeholders regarding the evaluation of peer mentorship programs in SCI community organizations, we conceptualized effectiveness indicators that would capture the extent that organizations were tracking outcomes, the methods they used to assess outcomes, and descriptions of both positive and negative outcomes reported by their members. Although these indicators did not allow us to measure a primary outcome relative to a public health goal [3], they did allow us to better understand how community-based organizations are measuring the effectiveness of their programs.

#### Adoption

When evaluating community-based programs, it may be irrelevant to include the typical adoption indicator to asses the number of organizations that have adopted a peer mentorship program given that the inclusion criteria required that organizations provide this program. Instead, it may be most appropriate to capture specific programmatic details that may differ between organizations. For the peer mentorship programs, we conceptualized adoption using two indicators to: 1) assess the setting in which peer mentorship is being offered and 2) learn how many organizations have adopted a formal training program for their peer mentorship staff. The first indicator allowed us to evaluate certain characteristics of the organizations that are providing peer mentorship [3], while the second provided important information pertinent for new organizations who wish to implement a peer mentorship program.

#### Implementation

Given our purpose was to use RE-AIM to evaluate the collective impact of several community-based programs, we did not include indicators that would assess specific adaptations that individual organizations have made to their program [3]. Contrary to most RE-AIM analyses [17], SCI organizations often track number of staff and staff time dedicated to the program and the costs of the programs. We were able to include these types of implementation indicators to compare the consistency of implementation across the organizations. For example, to address staff implementation, we evaluated the percentage of staff that were specifically dedicated for peer mentorship at each of the nine organizations. Moreover, we evaluated the number of current staff dedicated to peer mentorship compared to the desired number that an organization would like to have. This indicator is unique to the community setting as research-based RE-AIM analyses typically know in advance the number of staff required to operate the intervention. Evaluating the cost of peer mentorship (time and money) was accomplished through two indicators: 1) how many full-time equivalent staff are dedicated to peer mentorship (time), and 2) what proportion of an organization’s budget is allocated to peer mentorship (money). Given that peer mentorship programs are on-going (no completion date), the above indicators were the only way to effectively conceptualize the cost of peer mentorship. Indicators to evaluate fidelity were not conceptualized given that: 1) fidelity checks are not common in community organizations due to resource constraints, and 2) organizations most likely deliver their programs differently from one another (i.e. varied delivery processes).

#### Maintenance

At the setting level, the length of time that an organization offers a program is one of the only indicators that can be collected, including in our study. A strength of this indicator is that it provides data on the extent to which the program has been institutionalized, a common limitation in research-based RE-AIM analysis. As it was in our study, evaluating maintenance at the individual level can be challenging in community settings as community-based programs may monitor effectiveness differently compared to typical research interventions. For our purposes, maintenance at the individual level comprised of two indicators for mentors (i.e., the number of peer mentors that joined the organization in the past 5 and 10 years) and three for mentees (i.e., the number of peer mentees that received mentorship in the past year, 5 years, and 10 years). The strength of these indicators is that they allowed us to evaluate the sustainability of peer mentorship from a participant enrollment perspective.

### Procedures

All procedures were approved by the research ethics board at The University of British Columbia (Okanagan Campus) before data collection. All organizations willing to participate completed a consent form administered by the lead researcher and were sent a link to an online survey. Survey questions were developed in collaboration between researchers and community stakeholders to ensure the questions were theoretically sound, practically meaningful for the end user (i.e., community SCI organizations), and targeted each of the RE-AIM dimensions. Stakeholders included the executive director of SCI Canada, as well as a current executive director of a provincial SCI organization. These stakeholders work closely with individuals with SCI and are extremely knowledgeable about the peer-mentorship programming currently being delivered in Canada.

The lead researcher contacted each organization two weeks after they received the survey link to answer questions and provide technical support. Organizations were not given a timeline to complete the survey but were encouraged to complete it at their earliest convenience without interrupting their day to day operations. On-going support was provided through email or telephone as needed until the survey was complete.

After completing the online survey, executive directors were contacted by the lead author to complete a short telephone interview regarding their participation in the study. To avoid limiting participant responses and encourage richer conversations, questions were open-ended, grouped around the five RE-AIM dimensions, and tailored based on the survey responses from each individual organization. Organizations were remunerated with an hourly wage based on the time it took them to complete the study.

### Measures

#### Online survey

Indicators specific to SCI peer mentorship were formulated for each of the five RE-AIM dimensions as outlined in Table 1. These indicators were then worded in the form of 48 open-ended questions that were administered through Fluid Surveys. Organizations took an average of 4 h to complete the survey. A complete list of the questions can be found in Additional file 1.

**Table 1:** RE-AIM Indicators, Numerators, and Denominators

RE-AIM Elements	Descriptions	Numerator	Denominator	Other
Reach *National* Peer Mentors	The percentage of Canadians with SCI who are peer mentors	Total number of registered peer mentors	All Canadians with SCI [28]	
Peer Mentor Representativeness	How representative registered peer mentors are compared to general Canadian SCI population			Demographic information of peer mentors compared to population estimates [28]
Peer Mentees	The percentage of Canadians with SCI who have received mentorship	Total number of Canadians with SCI who have received mentorship	All Canadians with SCI [28]	
Peer Mentee Representativeness	How representative are people who have received mentorship compared to general Canadian SCI population			Demographic information of peer mentees compared to population estimates [28]
*Provincial* Peer Mentors	The percentage of persons with SCI who are registered peer mentors in each province	Total number of registered peer mentors in a province	Total number of Canadians with SCI in the province [28]	
Peer Mentees	The percentage of persons with SCI who received mentorship in each province	Total number of people who have received mentorship in a province	Total number of Canadians with SCI in the province [28]	
Effectiveness *Provincial* Peer Mentor Outcome	The reported positive and negative outcomes of engaging in mentoring for peer mentors			Qualitative data from surveys
Peer Mentee Outcomes	The reported positive and negative outcomes of engaging in mentoring for peer mentees			Qualitative data from surveys
Adoption *Setting Level* Peer Mentor Programming	The % of organizations providing peer mentoring	The # of SCI organizations providing peer mentoring	The # of SCI organizations surveyed	
Peer Mentor Programming – community	The % of organizations who provide community peer mentoring	The # of SCI organizations providing peer mentorship in the community	The # of SCI organizations surveyed	
Peer Mentor Programming – hospital	The % of organizations who provide inpatient peer mentoring	The # of SCI organizations providing inpatient peer mentorship	The # of SCI organizations surveyed	
Hospital adoption of peer mentoring	The % of hospitals where peer mentoring is occurring	The # of hospitals where inpatient mentoring is provided by a SCI organization	The # of SCI rehabilitations hospitals in Canada	
Peer Mentoring Branches	The # of branches of peer mentoring services within each provincial organization			Information collected from surveys (# of reported peer mentoring branches)
Implementation *National Level* Outcome data collection	The % of organizations that collect outcome data	The # of organizations that collect outcome data	The total # of SCI organizations	
On-going Training	The % of organizations that provide continued/on-going training	The # of organizations that offer on-going training	The total # of organizations that offer training	
Monitoring Practices	The % of organizations that monitor their mentor/mentee relationships	The # of organizations that track mentor-mentee interactions	The total # of SCI organizations	
*Provincial Level* Cost of Service	The % of money dedicated to peer mentoring	The amount of money allocated for peer mentorship program	Total amount of money in operation budget of an organization	
Cost per member	Operational budget cost per mentor	The amount of money allocated for peer mentorship program	Total number of registered mentors in an organization	
Peer mentors as intended	The % of peer mentors in the organization as intended	The total # of registered peer mentors in an organization	The # of peer mentors an organization wants available	
Program efficacy	The % of mentors who actually end up mentoring	The # of mentors who have mentored someone	The # of mentors who have been trained	
*Staff Level* Peer mentoring staff	The % of staff dedicated to peer mentoring	The # of staff employed for peer mentoring (FTE equivalent)	The # of staff employed by an organization	
Peer mentoring staff as intended	The % of staff in an organization dedicated to peer mentoring as intended	The # of staff employed for peer mentoring (FTE equivalent)	The # of staff (FTE equivalent) an organization wants available for peer mentoring	
Peer mentoring volunteers	The % of volunteers dedicated to peer mentoring	The # of volunteers dedicated to peer mentoring	The # of volunteers employed by an organization	
Maintenance *Setting Level* Long term sustainability	The long-term sustainability of the program			How long the program has been operating
*Individual Level* Growth of peer programming – Mentor	The growth of the peer mentoring program within an organization	The # of registered peer mentors in the organization	The # of registered peer mentors in the organization 5 and 10 years ago	
Growth of peer programming – Mentee	The growth of the peer mentoring program within an organization	The # of people who have received mentorship in the past year	The # of people who received mentorship 5 and 10 years ago	

#### Telephone interview

The interview schedule (Additional file 2) was developed by two of the authors and a stakeholder with the intent to a) further probe the answers provided in the online survey, and b) elicit ideas for future research related to SCI peer mentorship. Interviews began with questions pertaining to respondents’ experience completing the survey and transitioned into specific questions related to the answers they provided. Interviews lasted, on average, 25–30 min. All executive directors were offered the opportunity to follow up with the interviewer if they had any further questions or concerns.

### Data analysis

The data analysis was performed by the first author using summary statistics (e.g., proportions, medians, frequencies) to address the study objectives. The numerators and denominators used in each of the RE-AIM calculations are described in Table 1. The telephone surveys were transcribed verbatim and used to help interpret responses and address missing data from the online survey.

## Results

### RE-AIM analysis of SCI peer mentorship programs

#### Reach

For the 2016/17 fiscal year, peer mentorship programs belonging to participating organizations reached 1.65% of the estimated number of people living with SCI in their home provinces (range = 0.6–3.5%) and 1.63% of the entire estimated Canadian SCI population [28]. Mentors currently belonging to organizations were representative of the general SCI population for gender (men = 64.8%, women = 35.2%) but were slightly overrepresented by people with tetraplegia (54.7%) compared to the general SCI population (tetraplegia = 43.6%, paraplegia = 56.4%). Mentees who had received mentorship were also representative of the general SCI population for gender (men = 59.9%, women = 30.7%) and for severity of injury (tetraplegia = 47.7%, paraplegia = 52.3%). A summary table outlines the reach results in more detail (see Additional file 3).

#### Effectiveness

A total of 67% of organizations tracked positive outcomes/outputs and 56% tracked negative/unintended outcomes of peer mentorship. Organizations tracked outcomes and outputs using various methods (e.g., surveys, testimonials) and had documented some of the unique benefits of engaging in peer mentorship for both mentors and mentees. Key positive outcomes reported by mentors included an improved sense of purpose and increased relatedness while negative outcomes included feeling helpless and tired. Positive outcomes for mentees included improved well-being, increased confidence, and an improved outlook on life while the only negative reported outcome was not feeling ready to receive mentorship. A summary table outlines the effectiveness results in more detail (see Additional file 4).

#### Adoption

Of the nine recruited organizations, 89% offered a formal peer mentor training program and 100% provided peer mentorship in both hospital and community settings. Peer mentorship was being provided at 41 hospitals across Canada and in hundreds of different communities. Although the exact number of communities served was not able to be calculated, there were a total of 39 official offices/locations between the nine responding provincial organizations that provided peer mentorship. A summary table outlines the adoption results in more detail (see Additional file 5).

#### Implementation

Organizations utilized their available peer mentors efficiently, despite operating with just 61.5% of their desired number of peer mentors: 96.5% of registered peer mentors (*n* = 454) had mentored someone with a SCI. During the 2016/2017 fiscal year just over 1.9 million dollars (Canadian) were allocated to the operation of peer mentorship programming across the nine organizations, which accounted for 10.4% of the total operation budget across the organizations. A total of 25.7 (full time equivalent) paid staff (i.e. paid peer mentors and other staff) were employed specifically for peer mentorship across the nine organizations, accounting for 9.8% of the total staff employed. However, this number is only 39.8% of the preferred number of staff (*n* = 57) that organizations would like to have available for peer mentorship. In addition, 374 volunteers were dedicated to peer mentorship accounting for 30.2% of the total number of volunteers (*n* = 1239) across the organizations. And finally, 55.6% of organizations tracked their mentor-mentee interactions/relationships (e.g., frequency of interactions, topics of discussion). A summary table outlines the implementation results in more detail (see Additional file 6).

#### Maintenance

All nine organizations maintained the delivery of their peer mentorship programming for many years (M = 46.4 years, SD = 27.4, R = 7–71) with 89% having offered programming for over 10 years. Peer mentor membership across all nine organizations continues to grow with an increase of 183 new peer mentors across the organizations in the past 5 years, accounting for 35% of the current membership. Organizations who were able to provide data (*n* = 7) have provided peer mentorship to a total of 3872 peer mentees over the past 5 years with 10% of these occurring during the past year. A summary table outlines the maintenance results in more detail (see Additional file 7).

## Discussion

The first purpose of this paper was to operationalize and apply the RE-AIM framework to evaluate community-based public health programming developed and delivered by multiple, autonomous community organizations. A second purpose was to apply RE-AIM to evaluate the impact of Canadian SCI peer mentorship programs. Operationalizing the RE-AIM framework to evaluate the impact of Canadian SCI peer mentorship programs proved to be challenging but nevertheless achievable. Collaborating with key community stakeholders throughout the study allowed us to conceive and define key peer mentorship indicators within each of the five RE-AIM dimensions which facilitated assessment of the impact of peer mentorship at both the individual and organizational level.

### Operationalizing the RE-AIM framework to evaluate multiple programs

This study provided a new way to conceptualize RE-AIM to evaluate similar community-based public health programs delivered by multiple autonomous community organizations. Operationalizing the framework for these purposes led to several challenges. First, determining universal indicators that were appropriate to evaluate all nine different programs proved to be difficult. Typically, when evaluating a single program or intervention using RE-AIM, the researcher is able to determine specific indicators that fully capture the components within each RE-AIM dimension. For example, Schwingel, Galvez, Linares, and Sebastiao [29] determined the effectiveness of a community health program for older Latinas using three separate indicators based on valid and reliable measures administered during the program. Due to the lack of program standardization across the nine organizations, we were only able to evaluate effectiveness using two general indicators (Table 1) that our stakeholders believed to be consistent across all organizations. In doing so, we were unable to evaluate the effectiveness of individual organizations whose effectiveness could potentially have been conceptualized using indicators unique to their program. This lack of program standardization affected the way we conceptualized the indicators for all five RE-AIM dimensions and demonstrates a challenge of using RE-AIM to evaluate more than a single autonomous community-based program.

Another challenge was the variability in the types and amount of data being collected by each peer mentorship program. Although we engaged two stakeholders (both SCI program executive directors) to help create questions that fellow organizations would likely have data for, we still only collected complete data for 25 of the 48 questions. Data availability will likely always be a challenge when using the RE-AIM framework in a community setting [19, 20, 30]. The engagement of stakeholders to inform the survey questions and the use of a telephone survey to further explore responses and missing data [17, 31] were crucial in ensuring that we collected as much useable data as possible.

Lastly, because this study attempted to operationalize the RE-AIM framework pragmatically in a community-based setting, the authors deliberately loosened the constraints regarding what constitutes fully-developed use of the framework [17]. In doing so, we conceptualized how to address certain RE-AIM criteria differently than how these criteria would be evaluated in a clinical or research setting. For example, Kessler and colleagues [17] recommend that an essential criterion for evaluating the implementation of a program is to assess the percentage of perfect delivery or calls completed. After discussions with our stakeholders, this criterion was conceptualized as the percentage of peer mentors who mentor after being trained. Organizations may not have a system for evaluating if a peer mentor is delivering their mentorship “perfectly” (i.e. fidelity) as this is not a realistic objective that a peer mentor can attain. Instead, the objective may be to have as many trained mentors providing high quality mentorship as possible. Furthermore, using “the percentage of mentorship sessions completed” in place of “percentage of calls completed” is not appropriate as there would be no valid denominator. These examples highlight the challenge of using the RE-AIM framework in a community-based setting as the distinction between internal program objectives and external evaluation criteria can be challenging [20]. Researchers should strive to include a breadth of evaluation indicators that evaluate both the internal and external validity of a program despite the challenges posed in community settings.

### Evaluating the impact of SCI peer mentorship programs

#### Reach

At first glance, the individual-level reach of peer mentorship programs appears to be low at just 1.65% of the estimated SCI population of the provinces where organizations are located. However, in comparison with other large community interventions and programs that have used RE-AIM as their evaluation framework, we see that the reach of peer mentorship programs is relatively high. For example, Walk Kansas [30], a state-wide physical activity initiative, was able to reach 1% of the total population of the counties where the program was delivered. Similarly, an obesity program for children in the city of London, Ontario [18] reported a reach of 0.7%, and a recent physical activity and healthy eating program delivered across Canada reported a reach of 0.45% [19]. Thus, the annual reach of peer mentorship programs is as good, if not better than other population level programs. Furthermore, the historical reach could be quite large given that some organizations have been providing programming for over 70 years.

Although we attempted to capture data that would allow for assessment of historical reach, many organizations did not have accurate data-tracking practices for several years, which inhibited our ability to state their true reach. Furthermore, we defined our reach indicator as the number of unique individuals who have delivered (mentors) or received (mentees) peer mentorship. Restricting our interpretation of reach to this sole indicator most likely led to an underestimation of the true reach of peer mentorship. Other potential reach indicators (e.g., the number of peer mentorship events that were delivered, the number of resources distributed by peer mentorship programs, the number of informal peer mentorship occurring at community events) could have provided a better representation of the impact of these programs, which highlights the importance of evaluating all activities of a program when using the RE-AIM framework [10]. Researchers should strive to include a breadth of evaluation indicators that evaluate both the internal and external validity of a program despite the challenges posed in community settings. Stakeholders, who are key-decision makers, should also be involved throughout the entire research process to ensure that all potential indicators are being captured and are meaningful to the organization/program [5, 17, 32].

#### Effectiveness

Although several positive outcomes for peer mentors (e.g., improved emotional health, improved sense of purpose, increased relatedness) and peer mentees (e.g., sport, recreational, and social participation, improved knowledge of resources, improved self-care) were described, only 67% of organizations reported systematic tracking of these outcomes. During the interviews, a lack of resources was commonly described as a barrier to tracking outcomes methodically. Interestingly, some organizations also reported that they simply don’t know what measures to use. These concerns directly align with the findings from a recent Canada-wide consultation process which identified that the measures currently used to examine the outcomes of SCI peer mentorship do not capture the impact of these programs [33]. Tracking effectiveness becomes burdensome and unproductive without reliable measures that capture the intricate and often subtle benefits of peer mentorship [26]. Assessing the individual-level impact of peer mentorship quantitatively (e.g., calculation of effect sizes) was therefore not possible. Rather, impact could only be inferred using the data collected through the interviews. This limitation highlights the importance of using diverse methodology when performing a RE-AIM analysis [17], especially in community settings where collecting valid and reliable quantitative outcome data may not be feasible [4].

Although this study was unable to calculate the population health impact of peer mentorship programs, the qualitative data do allow for some discussion of the potential impact. For example, mentees indicated that a major benefit of engaging in peer mentorship was sport and recreational participation. Given that individuals with SCI are considered the least active segment of the population [34, 35], interventions/programs, like peer mentorship, that encourage and facilitate participation are critical. Based on recent economic evaluations [36], regular participation in physical activity by people with SCI can result in a cost savings of US$290,000–$435,000 over a lifetime, primarily due to fewer hospitalizations and less reliance on assistive care. Another benefit of peer mentorship listed by mentees was improved self-care. A recent intervention demonstrated significantly reduced re-hospitalization rates for people with SCI as a result of improved self-care learned by participating in peer mentorship [24]. Houlihan and colleagues [23] also demonstrated significant improvements in self-care management for people with SCI after engaging in a peer mentorship intervention. These findings are important given that the costs associated with SCI-related secondary conditions (e.g., pressure ulcers) can be upwards of CND$4745 per person, per month [37]. Taken together, the potential cost-savings associated with better SCI self-care are significant. As over one thousand Canadians receive mentorship through these organizations each year, the potential population health and economic impacts of Canadian SCI peer mentorship programs are likely profound.

#### Adoption

Given our inclusion criteria, it was unsurprising that 100% of organizations provided peer mentorship in both community and hospital settings. Of particular interest, was the number of hospitals where peer mentorship is occurring. Across Canada, there are 16 official SCI rehabilitation hospitals [38] yet peer mentorship is occurring in at least 41 hospitals, as indicated by the eight organizations who provided these data. This finding is encouraging as it suggests that community organizations are communicating and working in collaboration with various types of hospitals (e.g., rehabilitation, general, convalescent) to ensure people with SCI who could benefit from peer mentorship are being reached and served.

Unfortunately, due to the lack of clarity in our community adoption question (Additional file 5), we were unable to calculate the adoption rate at the community level. This oversight could have been avoided by providing definitions of ‘community’ to the participating organizations or eliciting greater feedback regarding the clarity of the questions from participants during the data collection process. Lastly, although not all organizations offer a formal peer mentor training program, there is not yet any clear consensus in the SCI literature as to what should be included in such a program [26, 39]. As such, it is difficult to formulate recommendations regarding peer mentorship training.

#### Implementation

Organizations are managing to reach a fair proportion of the SCI population despite only operating with 39.8% of their preferred number of peer mentorship specific staff. Burnout among staff of nonprofit organizations has been well documented as serving one’s community can come at the price of great personal sacrifice [40, 41]. Peer-mentorship programs across Canada must continue to monitor the physical and mental health of their staff to mitigate the negative outcomes associated with providing peer mentorship (i.e. feeling tired and hopeless). One way to potentially mitigate the risk of burnout is for organizations to allocate more funding towards their peer mentorship programs. Currently there is a large range in the amount of money allocated to the operation of peer mentorship programs with one organization allocating almost 46% of their operating budget while five others allocated between just 0–5%. Interestingly, despite this large range, the 2016/2017 peer mentee reach of organizations within their own home provinces did not vary greatly (range = 0.3–3.5%). The large number of volunteers dedicated to peer mentorship could explain how certain organizations are providing peer mentorship despite allocating minimal amounts of their operating budget and having less than the preferred number of staff. Calculating the economic value that peer mentor volunteers have on their respective organizations would be challenging [42] but warrants consideration for future research.

#### Maintenance

Despite each organization allocating different percentages of their funding to operate their peer mentorship program, each has been able to sustain the delivery of their programs for many years. Furthermore, all organizations have been able to sustain their programing despite having different service delivery models (e.g., one-to-one mentorship versus group-based mentorship, focus on mentorship in community versus hospital settings). These findings demonstrate that long-term maintenance of peer-mentorship programming is not restricted to a specific delivery model. This observation is encouraging given that an organization’s mandate may change depending on their funding [43]. For example, one organization indicated that their service delivery model (i.e. one-to-one mentorship focusing on employment) was directly influenced by the requirements outlined by their main funding source. However, if their funding source was different, they would offer more group-based mentorship opportunities and place less focus on employment. Further research needs to be conducted to evaluate the cost effectiveness of different peer mentorship service delivery models and the impact they have on the maintenance as well as the individual-level reach of a program.

### Recommendations for using the RE-AIM framework

Based on the challenges experienced during this study, we have provided the following recommendations for researchers to consider when applying the RE-AIM framework to evaluate the impact of real-world, community-based, public health programming developed and delivered by autonomous community organizations.

- Involve stakeholders from all participating organizations throughout the entire research process. Conceptualizing indicators that are reflective of the available data can only be accomplished by understanding the data monitoring methods of each organization. Furthermore, continuing to engage stakeholders during data collection can help inform qualitative follow-up questions to ask about the data that are being collected which will help facilitate a deeper understanding of results. Engaging with stakeholders will also allow you to facilitate conversation between organizations which could lead to collaborative health promoting initiatives between organizations. For example, organizations that serve people with chronic conditions (e.g. heart disease, diabetes) may choose to deliver similar health promoting initiatives (e.g. awareness runs) simultaneously to a) increase the potential reach of the initiative and b) reduce the financial cost to deliver the event.
- Understand the funding model used by each organization and how it impacts their programming. Every non-profit organization will have its own unique funding model that typically includes funding from a variety of sources (e.g., donations, grants, fundraising). The outcomes and outputs that an organization delivers and monitors are often dictated to them based on the funding they receive. Thus, the data that are collected and the programs that individual organizations provide will likely vary. Having a complete understanding of the funding model for all participating organizations will aid in the development of indicators that are universal across those organizations. This recommendation is imperative if using RE-AIM to evaluate health promotion programming delivered by a combination of non-profit and for-profit organizations. For example, a for-profit gym may have more funding and staff available to track specific outcomes for a certain exercise class compared to a non-profit gym that may not have the resources to collect robust data. Understanding these funding differences would be crucial to develop indicators that are meaningful and relevant for both exercise programs.
- Include indicators that may only be applicable to some programs. Evaluating the cumulative impact of several programs would require that all organizations collect the exact same data. As this may not always be the case, it is important not to exclude indicators that could still be used to evaluate the impact of some of the programs. For example, one organization may evaluate reach by examining the number of new members in a given year while the others may evaluate reach based on the number of resources distributed to the community. These sets of data could not be amalgamated, thus restricting our ability to evaluate the cumulative impact of these programs. However, the results from both reach indicators could still be compared, and a further understanding of why programs collect different data could be achieved through qualitative methods. Unique findings from one organization may be insightful for others about how they can improve their reach, monitor effectiveness, or implement their health promoting programs more effectively.
- When conceptualizing indicators for each RE-AIM dimension it is important to not only consider individuals that may be receiving services from a program but also those who are providing the program, especially if they are members of that community organization. Careful consideration must be given when conceptualizing indicators to ensure that all potential “participants” are considered. For example, when using RE-AIM to evaluate a community-based summer camp as Burke and colleagues did [18], it may be prudent to conceptualize reach indicators for camp counsellors in addition to campers, especially if counsellors belong to the same organization.

## Conclusion

This study helps extend the literature on the pragmatic uses of the RE-AIM framework by demonstrating a unique application of the framework in a community setting. Through the application of RE-AIM, we were able to provide preliminary information regarding the impact of SCI peer mentorship programs across Canada at both the individual and organizational level. Furthermore, this research has highlighted challenges with implementing the RE-AIM framework to evaluate community-based public health programming across multiple organizations and demonstrates the importance of involving stakeholders and using diverse methodology to account for missing, inconsistent, or unavailable data. The findings suggest that a better understanding of how to effectively use RE-AIM to evaluate programming delivered by multiple autonomous community organizations is needed. Researchers are encouraged to continue using the framework to evaluate the collective impact of multiple public health programs but should continue to report recommendations to iteratively improve our understanding of how to use RE-AIM in this context.

## Additional files

Additional file 1: RE-AIM Peer Mentoring Survey. (DOCX 26 kb)

Additional file 2: Telephone Interview Questions. (DOCX 15 kb)

Additional file 3: Comprehensive results for Reach. (DOCX 18 kb)

Additional file 4: Comprehensive results for Effectiveness. (DOCX 15 kb)

Additional file 5: Comprehensive results for Adoption. (DOCX 15 kb)

Additional file 6: Comprehensive results for Implementation. (DOCX 16 kb)

Additional file 7: Comprehensive results for Maintenance. (DOCX 15 kb)

## Data Availability

The datasets used and/or analysed during the current study are available from the corresponding author on reasonable request.

## Abbreviations

RE-AIM: Reach, Effectiveness, Adoption, Implementation, Maintenance
SCI: Spinal Cord Injury
