# Supplementary material for: Operationalizing the reach, effectiveness, adoption, implementation, maintenance (RE-AIM) framework to evaluate the collective impact of autonomous community programs that promote health and well-being
Source: BMC Public Health. 2019 Jun 24;19:803. doi: 10.1186/s12889-019-7131-4 (PMC6591988; doi:10.1186/s12889-019-7131-4)
Supplement: Supplementary file 1 — RE-AIM Peer Mentoring Survey. (DOCX 26 kb) [file 12889_2019_7131_MOESM1_ESM.docx]

**RE-AIM Peer Mentoring Survey**

**Applying the RE-AIM framework to evaluate the impact of Canadian spinal cord injury peer mentoring programs**

Instructions: This survey must be completed within the next 30 days. To save your responses and continue the survey at a later time click the "save and continue later" button located at the bottom of each survey page. Response fields are not restricted in size so please expand on your answers when necessary. We thank you again for participating in this important research study.

Participation or Withdrawal: Your organization’s participation in this study is voluntary. You may decline to answer any question and you have the right to withdraw from participation at any time. Withdrawal will not affect your relationship with the University of British Columbia or Spinal Cord Injury Canada in anyway. If you choose to not participate after volunteering for the study we ask that you email the student investigator. If you choose to withdraw from this study your completed data may still be analyzed. If you do not want to receive any more reminders, you may contact the research team at shawrb1@mail.ubc.ca or (705)-477-5205.

Contacts: If you have any concerns or complaints about your rights as a research participant and/or your experiences while participating in this study, contact the Research Participant Complaint Line in the UBC Office of Research Ethics at 604-822-8598 or if long distance e-mail RSIL@ors.ubc.ca or call toll free 1-877-822-8598.

**Section 1a: Reach**

**The following questions will help us better understand the absolute number, proportion, and representativeness of individuals who participate in your peer mentoring programs.**

1. How many registered peer mentors belong to your organization?
2. How many paid peer mentors belong to your organization?
3. How many volunteer peer mentors belong to your organization?
4. How many people with a spinal cord injury have received mentorship through your organization?

**Section 1b: Reach**

**The following questions pertain to the demographics of the registered peer mentors in your organization**

1. Age: Please provide the total # of mentors for each of the below age ranges:
2. Ethnicity: Please provide the total # of mentors who identify with each of the following ethnicities: White, Native Canadian, Black, Asian, Other
3. Gender: Please provide the total # of mentors who identify with each of the following genders: Male, Female
4. Disability type: Please provide the total # of mentors with a spinal cord injury who identify with the following injury levels: Quadriplegic/Tetraplegic (C1-C7), Paraplegic (T1-S5)
5. Disability type: Does your organization have peer mentors who identify as having a disability other than a spinal cord injury?

If yes,

1. How many peer mentors identify as having a disability other than a spinal cord injury?
2. Please list the other reported types of disability (e.g. muscular sclerosis)
3. Educational Level: Please provide the total # of mentors who have completed the following levels of education: High school, college, University, Postgraduate, Other
4. Marital Status: Please provide the total # of mentors who identify with each marital status: Single, Married, Divorced, Common Law, Widowed

**Section 1c: Reach**

**The following questions pertain to the demographics of the peer mentees who have received mentorship through your organization**

1. Age: Please provide the total # of mentees for each of the below age ranges:
2. Ethnicity: Please provide the total # of mentees who identify with each of the following ethnicities:  White, Black, Native Canadian, Asian, Other
3. Gender: Please provide the total # of mentees who identify with each of the following genders: Male, Female
4. Disability type: Please provide the total # of mentees with a spinal cord injury who identify with the following injury levels: Quadriplegic/Tetraplegic (C1-C7), Paraplegic (T1-S5)
5. Disability type: Does your organization have peer mentees who identify as having a disability other than a spinal cord injury?

If yes,

1. How many peer mentees identify as having a disability other than a spinal cord injury?
2. Please list the other reported types of disability (e.g. muscular sclerosis)
3. Educational Level: Please provide the total # of mentees who have completed the following levels of education: High school, college, university, postgraduate, other
4. Marital Status: Please provide the total # of mentees who identify with each marital status: single, married, divorced, common law, widowed.

**Section 2: Effectiveness/Efficacy**

**The following questions will assess the impact of your peer mentoring program at the individual level.**

1. Does your organization track outcomes or outputs of peer mentoring?

If yes,

1. How do you track outcomes (e.g., testimonials, reports, interviews, etc)
2. What are the reported outcomes for peer mentees? (e.g. return to work, increased social participation, better self-efficacy)
3. What are the reported outcomes for peer mentors? (e.g. feeling useful, increased happiness/satisfaction, improved relatedness)
4. Does your organization track negative or unintended outcomes associated with peer mentorship for peer mentors?

If yes,

1. List the negative or unintended effects of peer mentorship reported by peer mentors.
2. Does your organization track negative or unintended outcomes associated with peer mentorship for peer mentees?

If yes,

1. List the negative or unintended effects of peer mentorship reported by peer mentees.

**Section 3: Adoption**

**The following questions will ask about the absolute number, proportion, and representativeness of the peer mentoring programs in your organization.**

1. How many offices/locations in your organization provide peer mentoring services?
2. Does your organization have a formal training program for mentors?
3. Does your organization provide peer mentoring services in or at hospitals?

If yes,

1. At how many hospitals do you provide peer mentoring services
2. Does your organization provide peer mentoring services in a community setting?

If yes,

1. In how many communities do you provide peer mentoring services?

**Section 4: Implementation**

**The following questions will assess the cost and staff it takes to operate your peer mentoring program.**

1. What is the total amount of money in your organization’s operation budget?
2. How much money is allocated for your peer mentorship programs/services?
3. How many staff belong to your organization?
4. How many staff (Full Time Equivalent) are employed for peer mentorship?
5. How many staff (Full Time Equivalent) would you like available for peer mentoring?
6. How many volunteers belong to your organization?
7. How many volunteers are dedicated to peer mentoring?
8. Does your organization track/monitor mentor-mentee interactions/relationships? (e.g. how frequently they are interacting, the topics they discuss)

If yes,

1. What type of information do you track/monitor?
2. Does your organization offer on-going training for peer mentors?
3. How many peer mentors have mentored someone with a spinal cord injury?
4. How many peer mentors would you like to have in your organization?

**Section 5: Maintenance**

**The following questions will assess the degree to which your peer mentoring program has been sustained over time.**

1. How long has your organization’s peer mentoring program been operating for?
2. How many peer mentors have joined the organization within the last 5 years?
3. How many peer mentors have joined the organization within the last 10 years?
4. How many people have received mentorship from your organization in the past year?
5. How many people have received mentorship from your organization in the past 5 years?
6. How many people have received mentorship from your organization in the past 10 years?
