# Supplementary material for: Operationalizing the reach, effectiveness, adoption, implementation, maintenance (RE-AIM) framework to evaluate the collective impact of autonomous community programs that promote health and well-being
Source: BMC Public Health. 2019 Jun 24;19:803. doi: 10.1186/s12889-019-7131-4 (PMC6591988; doi:10.1186/s12889-019-7131-4)
Supplement: Supplementary file 2 — Telephone Interview Questions. (DOCX 15 kb) [file 12889_2019_7131_MOESM2_ESM.docx]

**Telephone Interview Questions**

**RECONFIRMING CONSENT**

Hello [NAME],

This is Rob, from UBC calling in regards to the SCI Peer Mentoring Study. Today you will participate in a telephone interview that will last about 15-30min.

Before we begin, I just want to confirm -that you still consent to participate?

Also please keep in mind you may withdraw from the study at any time without consequences. You may exercise the option of removing your data from the study. You may also refuse to answer any questions you don’t want to answer and still remain in the study.

The interview will be recorded so I will be putting you on speaker phone now.

Questions

1. Please discuss your experience completing the SCI peer mentoring surveying (e.g. discuss the challenges, why things were challenging, what could have made it easier)
2. After completing this study are there any changes you would like to make to your peer mentoring programming (e.g. services provided, data tracking methods, measures to track outcomes)
3. In your survey response I noted that your organization does not [ASK QUESTIONS REGARDING MISSING DATA]. Is there a reason why your organization does not collect this information (e.g. not enough resources to, unsure how to collect the data, or what data to collect)?
4. I also noted that you do not [ASK QUESTION REGARDING MISSING DATA]? Is there a reason why you do not collect this information? (e.g. not enough resources to, unsure how to collect the data,)?
5. Is there anything that your organization currently does not have that would benefit your peer mentoring programming (e.g. funding, more staff, etc)
6. With regard to your peer mentoring program, are there any services that you currently do not offer that you would like to? (if yes, what is preventing you from providing these services)
7. What areas of peer mentoring do you think future research should focus on? (e.g. outcomes, cost benefit, mentor training, etc)
8. How is your organization funded? (e.g. government, private, community, private donors, etc)

Alright well that’s all the questions that I have for you today. Do you have any questions for me?

Well thank you again for participating in this research study. I will keep you updated with the analysis and provide you with a report once it has been completed.

Take care
