# Supplementary material for: Operationalizing the reach, effectiveness, adoption, implementation, maintenance (RE-AIM) framework to evaluate the collective impact of autonomous community programs that promote health and well-being
Source: BMC Public Health. 2019 Jun 24;19:803. doi: 10.1186/s12889-019-7131-4 (PMC6591988; doi:10.1186/s12889-019-7131-4)
Supplement: Supplementary file 3 — Comprehensive results for Reach. (DOCX 18 kb) [file 12889_2019_7131_MOESM3_ESM.docx]

Additional file 3. Comprehensive results for Reach

| **Reach** |  |  |  |
| --- | --- | --- | --- |
| Original Research Question | # of responding organizations | Results | Comments on missing data |
| 1. How many registered peer mentors belong to your organization? | N=9 | Median=25; R=10-250 |  |
| 2. How many paid peer mentors belong to your organization? | N=9 | Median=3; R=0-13 |  |
| 3. How many volunteer peer mentors belong to your organization | N=8 | Median=11; R=7-241 | - Uncertain record keeping |
| 4. Please provide the following demographic information for the registered peer mentors in your organization.  a) Age    b) Ethnicity    c) Gender  d) Disability type    e) Education Level      f) Marital Status | N=7  N=8  N=9  N=9  N=4  N=6 | Age 10-19 (2.3%)  Age 20-29 (10.2%)  Age 30-39 (15.9%)  Age 40-49 (32.9%)  Age 50-59 (20.5%)  Age 60-69 (10.2%)  Age 70+ (7.9%)  White (90.6%)  Native Cnd (5.4%)  Black (0.5%)  Asian (1%)  Other (2.5%)  Male (64.8%)  Female (35.2%)  Other (0%)  Tetraplegic (54.7%)  Paraplegic (45.3%)  High School (30.4%)  College (31.6%)  University (25.3%)  Post Graduate (11.4%)  Other (12.3%)  Single (34.1%)  Married (40.5%)  Divorced (12.7%)  Common Law (9.5%)  Widowed (3.2%) | - Organizations do not collect this information  - Only have a rough average  - Only have partial data (i.e. less than 40% of current membership)  - Organizations do not collect this information  - Organizations do not collect this information |
| 5. How many people with a SCI have received mentorship through your organization? | N=1 | Total=150 | -Uncertain record keeping (e.g., some organizations go back 71 years)  -Provided the # of non-unique interactions  -Question was too vague (i.e., didn’t specify the timeframe) |
| 6. How many people with a SCI have received mentorship through your organization in the past year? | N=8 | Median=32; R=10-176 | -Provided the # of non-unique interactions (i.e. impossible to know how many unique individuals were served) |
| 7. Please provide the following demographic information for the peer mentees who have received mentorship through your organization.  a) Age    b) Ethnicity    c) Gender    d) Disability type    e) Education Level    f) Marital Status | N=6  N=5  N=6  N=6  N=3  N=6 | Age 10-19 (1.8%)  Age 20-29 (14.6%)  Age 30-39 (19.2%)  Age 40-49 (16.6%)  Age 50-59 (20.4%)  Age 60-69 (16.9%)  Age 70+ (10.3%)  White (82.3%)  Native Cnd (10.4%)  Black (0%)  Asian (1.7%)  Other (5.6%)  Male (59.9%)  Female (30.7%)  Other (9.3%)  Tetraplegic (47.7%)  Paraplegic (52.3%)  High School (57%)  College (31.2%)  University (9.9%)  Post Graduate (0.6%)  Other (1.3%)  Single (36.1%)  Married (38.4%)  Divorced (12.5%)  Common Law (10.1%)  Widowed (2.9%) | - Organizations do not collect this information  - Only provided a rough average  - Organizations do not collect this information  - Only have partial data  - Organizations do not collect this information  - Only provided rough percentages  - Organizations do not collect this information  - Only provided a rough average  - Organizations do not collect this information  - Organizations do not collect this information |
