# Supplementary material for: Operationalizing the reach, effectiveness, adoption, implementation, maintenance (RE-AIM) framework to evaluate the collective impact of autonomous community programs that promote health and well-being
Source: BMC Public Health. 2019 Jun 24;19:803. doi: 10.1186/s12889-019-7131-4 (PMC6591988; doi:10.1186/s12889-019-7131-4)
Supplement: Supplementary file 4 — Comprehensive results for Effectiveness. (DOCX 15 kb) [file 12889_2019_7131_MOESM4_ESM.docx]

Additional file 4. Comprehensive results for Effectiveness

| **Effectiveness** |  |  |  |
| --- | --- | --- | --- |
| Original Research Question | # of responding organizations | Results | Comments on missing data |
| 1. Does your organization track outcome/outputs of peer mentoring?  a) If yes, How do you track outcomes?  b) If yes, What are the reported outcomes for peer mentors?    c) If yes, what are the reported outcomes for peer mentees?    2. Does your organization track negative or unintended outcomes for peer mentors?  a) If yes, what are the reported negative or unintended effects?  3. Does your organization track negative or unintended outcomes for peer mentees?  a) If yes, what are the reported negative or unintended effects? | N=9  N=6  N=6  N=5  N=9  N=5  N=9  N=5 | Yes=6, No=3  Testimonials, surveys, reports, interviews  Improved emotional health, improved sense of purpose, increased relatedness, improved motivation and self-confidence,  Sport/recreational participation, social participation, improved well-being, improved adjustment to SCI, increased confidence, improved knowledge of resources, increased relatedness, improved outlook on life, improved self-care, feeling supported  Yes=5, No=4  Feeling helpless, feeling tired, feelings of failure  Yes=5, No=4  Feeling not ready for mentorship | - do not have the means/resources to collect this data |
