# Supplementary material for: Operationalizing the reach, effectiveness, adoption, implementation, maintenance (RE-AIM) framework to evaluate the collective impact of autonomous community programs that promote health and well-being
Source: BMC Public Health. 2019 Jun 24;19:803. doi: 10.1186/s12889-019-7131-4 (PMC6591988; doi:10.1186/s12889-019-7131-4)
Supplement: Supplementary file 5 — Comprehensive results for Adoption. (DOCX 15 kb) [file 12889_2019_7131_MOESM5_ESM.docx]

Additional file 5. Comprehensive results for Adoption

| **Adoption** |  |  |  |
| --- | --- | --- | --- |
| Original Research Question | # of responding organizations | Results | Comments on missing data |
| 1. How many offices/locations in your organization provide peer mentoring services?  2. Does your organization provide peer mentoring services in or at hospitals?  a) If yes, at how many hospitals do you provide peer mentoring services?  3. Does your organization provide peer mentoring services in a community setting?  a) If yes, in how many communities do you provide peer mentoring services?  4. Does your organization have a formal training program for mentors? | N=9  N=9  N=8  N=9  N=4  N=9 | Median=2; R=1-10  Yes=9, No=0  Median=3.5, R=1-20  Yes=9, No=0  Median=5; R=1-9  Yes=8, No=1 | -The exact number of hospitals is unknown as the organization does not track their mentor-mentee interactions  -Question was too vague (i.e. what constitutes a community?)  -Data tracking would be to be involved (e.g., services provided across entire provinces) |
