# Supplementary material for: Operationalizing the reach, effectiveness, adoption, implementation, maintenance (RE-AIM) framework to evaluate the collective impact of autonomous community programs that promote health and well-being
Source: BMC Public Health. 2019 Jun 24;19:803. doi: 10.1186/s12889-019-7131-4 (PMC6591988; doi:10.1186/s12889-019-7131-4)
Supplement: Supplementary file 6 — Comprehensive results for Implementation. (DOCX 16 kb) [file 12889_2019_7131_MOESM6_ESM.docx]

Additional file 6. Comprehensive results for Implementation

| **Implementation** |  |  |  |
| --- | --- | --- | --- |
| Original Research Question | # of responding organizations | Results | Comments on missing data |
| 1. What is the total amount of money in your organization’s operation budget?  2. How much money is allocated for your peer mentorship programs/services?  3. How many staff belong to your organization?  4. How many staff (Full Time Equivalent) are employed for peer mentorship?  5. How many staff (Full Time Equivalent) would you like available for peer mentoring?  6. How many volunteers belong to your organization?  7. How many volunteers are dedicated to peer mentoring?  8. Does your organization track/monitor mentor-mentee interactions/relationship?  a) If yes, what type of information do you track/monitor?  9. Does your organization offer on-going training for peer mentors?  10. How many peer mentors have mentored someone with a spinal cord injury?  11. How many peer mentors would you like to have in your organization? | N=9  N=9  N=9  N=9  N=7  N=8  N=9  N=9  N=5  N=9  N=9  N=9 | Median=$2,088,000; R=$600,000-$9,919,000  Median=$90,000; R=$0-$550,000  Median=15; R=1-156  Median=1; R=0-8  Median=3; R=1-20  Median=63.5; R=18-446  Median=11; R=0-241  Yes=5, No=4  number of interactions,  duration of interactions,  number of one-to-one matches,  topics discussed,  method of interaction (phone, email, in person etc),  Mileage between mentor and mentee  Yes=6, No=3  Median=25; R=4-250  Median=34; R=3-500 | -Organizations were unable to provide an accurate number  -Only provided a rough number (i.e. >100) |
