# Supplementary material for: Operationalizing the reach, effectiveness, adoption, implementation, maintenance (RE-AIM) framework to evaluate the collective impact of autonomous community programs that promote health and well-being
Source: BMC Public Health. 2019 Jun 24;19:803. doi: 10.1186/s12889-019-7131-4 (PMC6591988; doi:10.1186/s12889-019-7131-4)
Supplement: Supplementary file 7 — Comprehensive results for Maintenance. (DOCX 15 kb) [file 12889_2019_7131_MOESM7_ESM.docx]

Additional file 7. Comprehensive results for Maintenance

| **Maintenance** |  |  |  |
| --- | --- | --- | --- |
| Original Research Question | # of responding organizations | Results | Comments on missing data |
| 1. How many years has your organization’s peer mentoring program been operating for?  2. How many peer mentors have joined the organization within the last 5 years?  3. How many peer mentors have joined the organization within the last 10 years?  5. How many people have received mentorship from your organization in the past 5 years?  6. How many people have received mentorship from your organization in the past 10 years? | N=9  N=8  N=7  N=6  N=4 | Median=60; R=7-71  Median=9; R=2-101  Median=12, R=7-337  Median=263; R=30-2708  Median=409; R=60-1006 | -Data tracking has been too inconsistent up until 2016 to provide an accurate number  -Data tracking has been too inconsistent up until 2016 to provide an accurate number  -Data was not available  -Uncertain record keeping  -Only provided a rough number (i.e. 20+)  - Provided the # of non-unique interactions (i.e. impossible to know how many unique individuals were served)  -Uncertain record keeping  -Provided the # of non-unique interactions (i.e. impossible to know how many unique individuals were served) |
